# Supplementary figures and images for: Effects of Stocking Density on Growth Performance and Stress Responses of Bester and Bester ♀ × Beluga ♂ Juveniles in Recirculating Aquaculture Systems
Source: Animals (Basel). 2021 Aug 3;11(8):2292. doi: 10.3390/ani11082292 (PMC8388450; doi:10.3390/ani11082292)

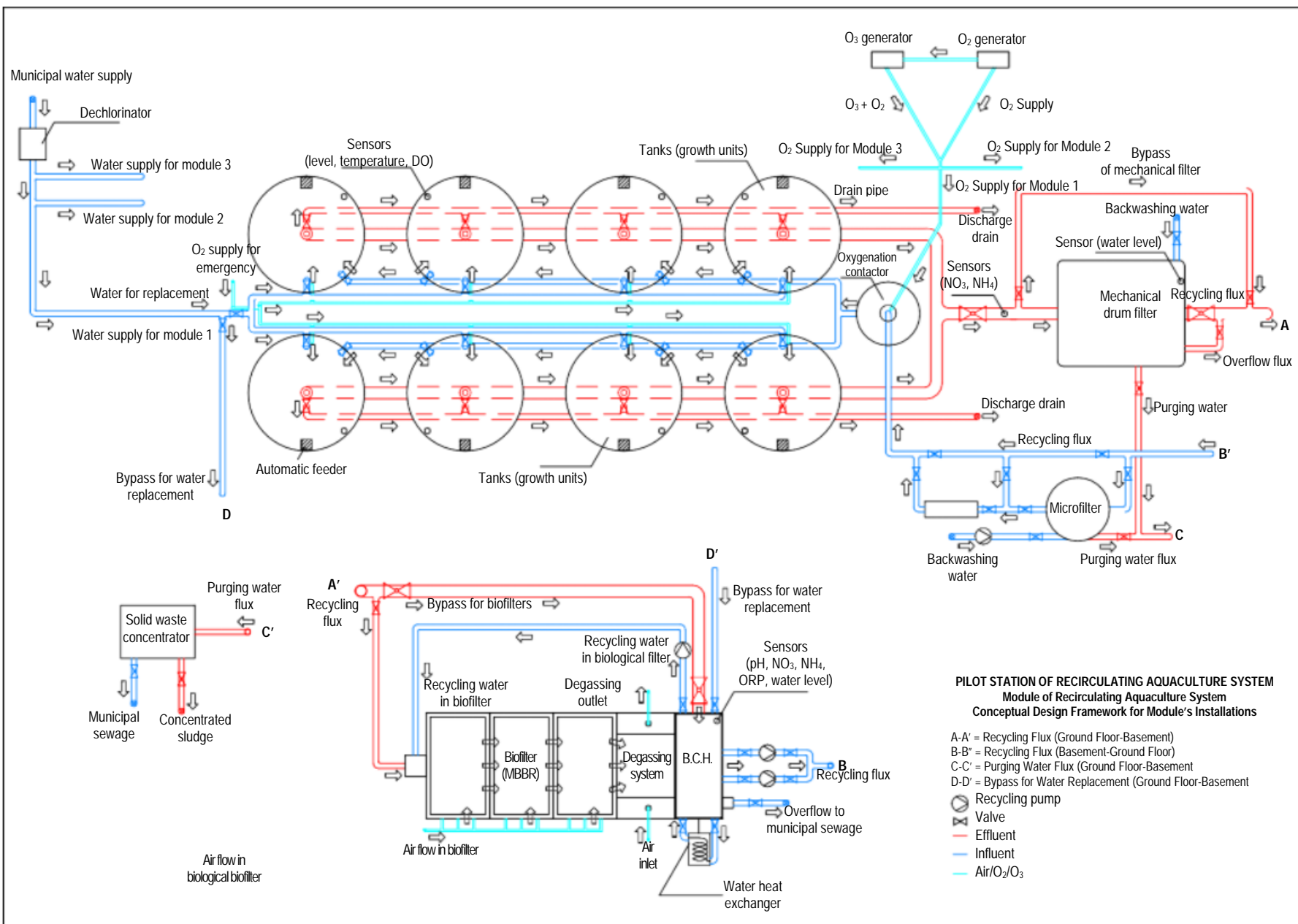

Supplement: Supplementary file 1 [file animals-11-02292-s001.zip › animals-1230190-supplementary.pdf]
